# Loss of Liver Kinase B1 (LKB1) in Beta Cells Enhances Glucose-stimulated Insulin Secretion Despite Profound Mitochondrial Defects*

**DOI:** 10.1074/jbc.M115.639237

**Published:** 2015-07-02

**Authors:** Avital Swisa, Zvi Granot, Natalia Tamarina, Sophie Sayers, Nabeel Bardeesy, Louis Philipson, David J. Hodson, Jakob D. Wikstrom, Guy A. Rutter, Gil Leibowitz, Benjamin Glaser, Yuval Dor

**Affiliations:** From the ‡ Department of Developmental Biology and Cancer Research, The Institute for Medical Research Israel-Canada, The Hebrew University-Hadassah Medical School, Jerusalem 91120, Israel,; § Department of Medicine, University of Chicago, Chicago, Illinois 60637,; ¶ Section of Cell Biology and Functional Genomics, Division of Diabetes Endocrinology and Metabolism, Department of Medicine, Imperial College London, SW7 2AZ, London, United Kingdom,; ‖ Massachusetts General Hospital Cancer Center, Harvard Medical School, Boston, Massachusetts 02114,; ** Endocrinology and Metabolism Service, Department of Internal Medicine, Hadassah-Hebrew University Medical Center, Jerusalem 91120, Israel, and; ‡‡ Unit of Dermatology and Venereology, Department of Medicine, Karolinska Institutet, Karolinska University Hospital, 171 77 Stockholm, Sweden

**Keywords:** calcium channel, insulin secretion, liver kinase B1 (LKB1), mitochondria, mitochondrial metabolism, pancreatic islet, *K*_ATP_ channel

## Abstract

The tumor suppressor liver kinase B1 (LKB1) is an important regulator of pancreatic β cell biology. LKB1-dependent phosphorylation of distinct AMPK (adenosine monophosphate-activated protein kinase) family members determines proper β cell polarity and restricts β cell size, total β cell mass, and glucose-stimulated insulin secretion (GSIS). However, the full spectrum of LKB1 effects and the mechanisms involved in the secretory phenotype remain incompletely understood. We report here that in the absence of LKB1 in β cells, GSIS is dramatically and persistently improved. The enhancement is seen both *in vivo* and *in vitro* and cannot be explained by altered cell polarity, increased β cell number, or increased insulin content. Increased secretion does require membrane depolarization and calcium influx but appears to rely mostly on a distal step in the secretion pathway. Surprisingly, enhanced GSIS is seen despite profound defects in mitochondrial structure and function in LKB1-deficient β cells, expected to greatly diminish insulin secretion via the classic triggering pathway. Thus LKB1 is essential for mitochondrial homeostasis in β cells and in parallel is a powerful negative regulator of insulin secretion. This study shows that β cells can be manipulated to enhance GSIS to supra-normal levels even in the face of defective mitochondria and without deterioration over months.

## Teaser

**Background:** LKB1 regulates multiple aspects of pancreatic β cell biology.

**Results:** LKB1 loss in β cells leads to profound mitochondrial defects yet increases glucose-stimulated insulin secretion in a mitochondria-independent mechanism.

**Conclusion:** LKB1 is essential for mitochondrial maintenance and negatively regulates a distal step of insulin secretion.

**Significance:** LKB1 loss exposes powerful mechanisms of insulin secretion that can override defects in the classic triggering pathway.

## Introduction

A large body of evidence has positioned LKB1^6^ as an evolutionarily conserved, central regulator of diverse cellular processes. LKB1 is essential for the determination and maintenance of proper cell polarity (1–3), for genome integrity and cytokinesis (4–6), and for linking cellular energy charge to cellular metabolism (7). All functions of LKB1 are believed to be mediated via phosphorylation and activation of a well defined set of 12 kinases from the AMPK family (8, 9). For example, phosphorylation of AMPK restricts mTOR signaling (via phosphorylation of TSC1/2) and lipid biosynthesis (via phosphorylation of acetyl-CoA carboxylase) (10–13), whereas phosphorylation of BRSK1/2 and MARK1–4 regulates the cytoskeleton, thereby determining cell polarity (14–16). At the tissue level, LKB1 functions as a powerful tumor suppressor gene (17–19) that is also essential for the maintenance of stem cells in a quiescent state (20–22).

In the context of pancreatic β cells, we (23, 24) and others (25) previously showed that LKB1 has multiple structural and functional roles. Deletion of LKB1 in adult β cells led to cellular hypertrophy (due to increased mTORC1 activity, secondary to inactivation of AMPK), increased β cell proliferation, dramatic alteration of β cell polarity (due to inactivation of MARK2), and increased glucose-stimulated insulin secretion, leading to accelerated clearance of glucose *in vivo* and to protection against high fat diet-induced glucose intolerance. The mechanisms underlying the enhancement of insulin secretion in LKB1-deficient β cells have remained ill-defined. It was proposed that altered polarity of β cells may enhance insulin secretion to nearby blood vessels (23) or alternatively that enhanced insulin secretion resulted from increased insulin content in β cells or increased overall β cell mass in LKB1 mutants (24, 25).

Recently, two direct phosphorylation targets of LKB1 were reported to act as positive regulators of glucose-stimulated insulin secretion. SIK2 was shown to enhance insulin secretion via phosphorylation and degradation of CDK5R1/p35 (26), and SAD-A was implicated as a regulator of β cell size and GSIS (27). Deletion of either gene disrupted insulin secretion. Because LKB1 deficiency is expected to functionally inactivate both SIK2 and SAD-A, a powerful mechanism must be activated upon LKB1 deletion that can compensate for these losses and lead to a net enhancement of insulin secretion.

Here we have examined the mechanisms accounting for enhanced insulin secretion in LKB1-deficient β cells. We demonstrate that enhanced secretion upon LKB1 inactivation requires the classical triggering pathway but acts primarily at a more distal step. Surprisingly, we found that LKB1 deficiency causes a dramatic deterioration of mitochondrial structure and function. However the amplification of insulin secretion by LKB1 deficiency overrides this defect, exposing a hitherto unrecognized mechanism for long term enhancement of β cell function.

## Experimental Procedures

### 

#### 

##### Mice

Strains used in this study were LKB1^lox/lox^ (2) crossed with either pdx1-CreER^TM^ (28), insulin-CreER^TM^ (29), or Ins1-Cre (30). These configurations resulted in essentially identical *in vivo* glucose homeostasis phenotypes (not shown and see Ref. 30). We encountered difficulties in islet isolation from Pdx1-CreER;LKB1^lox/lox^ mice after tamoxifen injection, probably due to acinar deletion of LKB1 that affected the islet mantle. Therefore, *in vitro* experiments were performed on islets isolated from Insulin-CreER;LKB1^lox/lox^ mice or Ins-Cre;LKB1^lox/lox^ mice. For convenience, LKB1-deficient mice are labeled in the manuscript as βLKB mice. Controls were lox/lox littermates.

Tamoxifen (Sigma, 20 mg/ml in corn oil) was injected subcutaneously to adult mice (1–2 months old). Two daily doses of 8 mg were used to achieve near total deletion of LKB1 in β cells, and animals were studied 2–16 months later. Because recombination occurred in utero in Ins1-Cre;LKB1^lox/lox^ mice (30), these animals were used at younger ages (8–12 weeks) as indicated in Fig. 4. Glyburide and Nifedipine were injected intraperitoneally at the indicated doses. Measurements of blood glucose and serum insulin were performed as described elsewhere (31). The joint ethics committee (Institutional Animal Care and Use Committees) of the Hebrew University and Hadassah Medical Center and the United Kingdom Home Office (PPL 70/06608) approved the study protocol for animal welfare. The Hebrew University is an AAALAC International-accredited institute.

##### Pancreatic Insulin Content and β Cell Mass

The pancreas was homogenized in acid ethanol (0.18 m HCl in 70% ethanol). After overnight incubation at 4 °C, homogenate was diluted 1:10 with 0.1% BSA in PBS. Insulin content was measured using the ELISA kit (Crystal Chem) and was calculated per pancreas weight.

For β cell mass calculation, consecutive paraffin sections 75 μm apart spanning the entire pancreas (∼10 sections/pancreas) were stained for insulin and hematoxylin. Digital images of sections at a magnification of ×40 were obtained and stitched using NIS-Elements software, and the fraction of tissue covered by insulin staining was determined. β cell mass was calculated as the product of pancreas weight and the fraction of tissue covered by β cells.

##### Isolation and Culture of Islets of Langerhans

Islets were isolated using collagenase P (Roche Applied Science) injected to the pancreatic duct followed by Histopaque (1119 and 1077, Sigma) gradient. Insulin secretion experiments were performed after an overnight incubation in RPMI 1640 supplemented with 10% fetal bovine serum, l-glutamine, and penicillin/streptomycin unless stated otherwise.

##### Adenoviral Infection of Islets

Islets were infected with an adenovirus encoding human LKB1 (Ad-STK11, Vector Biolabs, Philadelphia, PA). Infection was performed at the multiplicity of infection indicated in RPMI 1640 treated with 100 units/ml penicillin and streptomycin and 10% (v/v) heat-inactivated fetal bovine serum (FBS) for 48 h before measurements.

##### Static and Dynamic Stimulation of Insulin Secretion

Insulin secretion studies were performed in Krebs-Ringer buffer (KRBB) containing 114.4 mmol/liter NaCl, 5 mmol/liter KCl, 24 mmol/liter NaHCO_3_, 1 mmol/liter MgCl_2_, 2.2 mmol/liter CaCl_2_, 10 mmol/liter HEPES, and 0.5% BSA, adjusted to pH 7.35. In static incubation experiments, 25–30 islets were preincubated in basal KRBB containing 2.8 mm glucose for 1 h. Islets were then consecutively incubated at 2.8 and 16.7 mm glucose for 1 h each. Medium was collected at the end of each incubation period. Insulin assays were performed in Eppendorf tubes at 37 °C and 5% CO_2_.

For dynamic assessment of insulin secretion, we used a perifusion system (Biorep) equipped with a peristaltic pump. Fifty size-matched islets were placed in columns and perifused at a flow rate of 100 μl/min with KRBB at 37 °C. Perifusion started with 2.8 mm glucose for equilibration and measurement of basal secretion and then exposed to different treatments. Medium was collected to 96-well plates, and insulin was measured by ELISA (Crystal Chem) and normalized to total islet DNA or protein as indicated. DNA was isolated using DNeasy Blood and Tissue kit (Qiagen).

##### Measurement of Intracellular Free Ca^2+^

Whole islets were loaded with fura-2AM (Invitrogen) for 15 min at 37 °C in KRBB containing 119 mmol/liter NaCl, 4.7 mmol/liter KCl, 2.5 mmol/liter CaCl_2_, 1.2 mmol/liter MgSO_4_, 1.2 mmol/liter KH_2_PO_4_, and 25 mmol/liter NaHCO_3_. Fluorescence imaging was performed using a CCD-based imaging system and MetaFluor software (Universal Imaging), whereas islets were kept at 37 °C and constantly perifused with KRBB containing 2.8 mm or 16.7 mm glucose at a flow rate of 2.5 ml/min. Intracellular Ca^2+^ was expressed as the ratio of fluorescence intensity (at 535/30 nm) after illumination at 340 and 380 nm. For each experiment we used 8–20 islets (32). In some experiments Ca^2+^ imaging was performed using the non-ratiometric trappable fluorescent dye fluo-2 and Nipkow spinning disc confocal microscopy as described before (33).

##### Measurement of Intracellular Glutamate

Intracellular glutamate was measured using Glutamate Assay Kit (Sigma, MAK004) per the manufacturer's instructions. We used 150 islets for each measurement. Calculated glutamate concentration was normalized to total protein content (measured by BCA kit, Pierce).

##### Mitochondrial Analysis

To measure mitochondrial membrane potential we used TMRE (tetramethylrhodamine, ethyl ester; Molecular Probes). Dissociated islet cells were plated 2 days before the experiment on poly-d-lysine-coated dishes. Cells were incubated for 1.5 h before imaging with TMRE (10 nm) and MitoTracker Green (100 nm) loaded together with the MDR inhibitor, verapamil (50 μm), at 11 mm glucose. Analysis was performed using confocal imaging after wash out of MitoTracker Green, and mitochondrial membrane potential changes were calculated as previously described (34).

NAD(P)H autofluorescence was measured using the same imaging system and culture conditions described for calcium. Autofluorescence derived from NADH was excited at 365 nm and measured at 495 nm in dye-free islets. 6–15 whole islets were used for each experiment. Fluorescence readings of each islet were normalized to the first reading and averaged for each mouse.

For ATP/ADP imaging, isolated islets were cultured for 48 h with adenovirus (∼100 multiplicity of infection) expressing Perceval (35, 36) in RPMI 1640 medium treated with 100 units/ml penicillin and streptomycin and 10% heat-inactivated FBS. Islets were then placed in a custom-manufactured 36 °C chamber (Digital Pixel) mounted on a Zeiss Axiovert microscope coupled to a Nipkow spinning disk head. Islets were kept at 36 °C and continuously perfused with bicarbonate buffer at 95% O_2_/CO_2_ (37). Results were normalized to the minimum fluorescence (Fmin).

For real-time measurements of mitochondrial oxygen consumption we used Seahorse XF analyzer (38). Islets (50/well) were placed in 24-well islet plates with unbuffered DMEM supplied with 1% FCS and 2.8 mm glucose at 37 °C without CO_2_. Islets were then incubated at high glucose (16.7 mm) followed by consecutive treatment with FCCP (carbonyl cyanide 4-(trifluoromethoxy) phenylhydrazone, 1 μm) and rotenone (5 μm) plus antimycin (5 μm). Oxygen consumption rate (OCR) was calculated by the XF analyzer AKOS algorithm and normalized to basal levels or to total protein content. Protein was extracted with radioimmune precipitation lysis buffer, and total protein content was determined by Pierce BCA kit (Thermo Scientific).

To measure mitochondrial DNA copy number, DNA was isolated from fresh whole islets or from fixed sorted β-cells by standard phenol/chloroform extraction and ethanol precipitation. Quantitative real-time PCR was used to evaluate the ratio between cytochrome *b* (mitochondrial) and Aprt or L1 repetitive element (nuclear) with the following primers: cytochrome *b*, 5′-GCAGTCATAGCCACAGCA TTT-3′ and 5′-AAGTGGAAAGCGAAGAATCG-3′; Aprt, 5′-GGGATATCTCGCCCCTCTT-3′ and 5′-CACTCGCCTGCGATGTAGT-3′; L1, 5′-GTTACAGAGACGGAGTTTGGAG-3′ and 5′-CGTTTGGATGCTGATTATGGG-3′.

##### Transmission Electron Microscopy

Pancreas was fixed with 4% paraformaldehyde and 2.5% glutaraldehyde (Electron Microscopy Sciences), post-fixed with 1% osmium tetroxide (Sigma), and dehydrated with increasing concentrations of ethanol followed by propylene oxide (Sigma). For embedding we used Agar 100 Resin (Agar Scientific). For imaging we used 80-nm sections stained with 5% uranyl acetate for 10 min followed by 10 min with lead citrate. Samples were visualized with a transmission electron microscope (Technai 12, Phillips) equipped with a MegaView II CCD camera. To assess structural defects in mitochondria, we determined for each EM-imaged mitochondrion whether it was swollen or had defective cristae. Docked granules were counted up to 200 nm from the plasma membrane and calculated as number of granules per membrane length.

##### Western Blot

Protein was extracted from fresh islets by radioimmune precipitation lysis buffer supplemented with the protease and phosphatase inhibitors leupeptin, aprotonin, and vanadate. Total protein was determined by Pierce BCA protein assay kit (Thermo Scientific). Antibodies used were mouse anti-β-actin 1:10,000 (Sigma) and rabbit anti cytochrome *c* 1:1000 (Cell Signaling).

##### Quantitative Real-time PCR

RNA was isolated and purified from fresh islets with TRI reagent (Sigma) and RNeasy micro kit (Qiagen). cDNA was synthesized using 200 ng of RNA by the High-capacity cDNA Reverse Transcription kit (Applied Biosystems). For quantitative real-time PCR we used SYBR Green mix (Quanta Biosciences) and the following primers: PGC1α, 5′-GAGCCGTGACCACTGACAA-3′ and 5′-TGGTTTGCTGCATGGTTCT-3′; PGC1β, 5′-ATCGGGGTCCACCTTGAA-3′ and 5′-GGGTCACAGTTCTGGTTTGC-3′; β-actin, 5′-CACAGCTTCTTTGCAGCTCCT-3′ and 5′-GTCATCCATGGCGAACTGG-3′. Reactions were performed in triplicate in 96-well plates using CFX96 real-time System (Bio-Rad).

##### Statistics

Statistical analyses were performed using unpaired two-tailed Student's *t* test. Data are presented as the mean ± S.E. (unless otherwise indicated). *, *p* < 0.05; **, *p* < 0.01; ***, *p* < 0.005; ns, *p* > 0.05.

## Results

### 

#### 

##### LKB1 Deficiency in β Cells Leads to Persistent Enhancement of Glucose-stimulated Insulin Secretion

**FIGURE 1.**
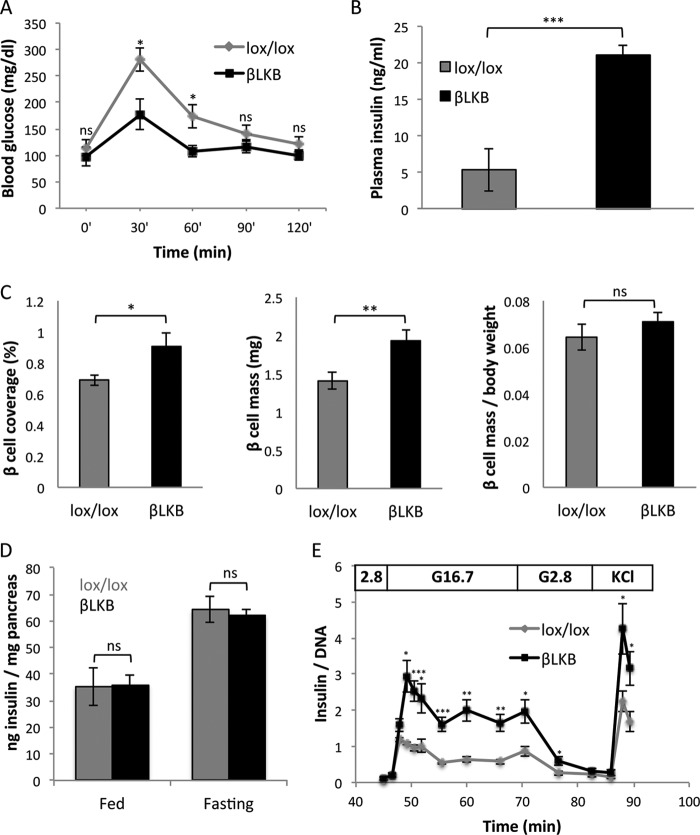
**Increased insulin secretion despite normal insulin content and β cell mass after LKB1 loss.** *A*, glucose tolerance test in old βLKB mice. Mice were injected with tamoxifen at 1 month of age, and assays were performed 16 months later. *n* = 4 or 5 per genotype. *p* value <0.05 by repeated measures analysis of variance. *B*, plasma insulin levels in old βLKB mice, 15 min after glucose injection. *Error bars* represent S.D. Mice were injected with tamoxifen at 1 month of age, and assay was performed 1 year later. *n* = 3 per genotype. *C*, β cell mass. Graphs represent the percentage of pancreas tissue area stained for insulin (*left*), the fraction of insulin-stained tissue multiplied by pancreas weight (total β cell mass in mg; center), and total β cell mass per body weight (*right*). Mice were 4 months old, 3 months after tamoxifen injection. *n* = 3 mice per group. *D*, insulin content in pancreata from fed and fasted lox/lox littermate controls and βLKB mice. Insulin content is presented relative to pancreas weight. βLKB mice do not differ in their pancreatic insulin content in either fed or fasted states. *n* = 4–7 mice per group. *E*, dynamic insulin secretion assay. Data represent the mean of data from 5 mice per group at age of 6–8 months. For each sample, measured insulin was normalized to total DNA. *, *p* < 0.05; **, *p* < 0.01; ***, *p* < 0.005; *ns*, *p* > 0.05.

We and others have previously shown that GSIS is enhanced after Cre-mediated deletion of LKB1 in β cells *in vivo* (23–25, 30). These observations were mostly obtained a short time after deletion of LKB1. To test if hyperfunctionality of β cells in βLKB mice declines with age, as seen in human type 2 diabetes and in some mouse models exhibiting enhanced insulin secretion (39), we measured glucose tolerance and serum insulin levels in βLKB mice up to 16 months after deletion. Injected glucose was cleared faster in mutant mice (Fig. 1*A*) along with greater insulin secretion (Fig. 1*B*). Thus deletion of LKB1 in β cells causes a persistent enhancement of β cell function.

Multiple mechanisms have been proposed to underlie increased insulin secretion in βLKB mice (23–25). We assessed β cell mass using morphometric analysis but found only a small increase in βLKB mice compared with controls (37% or less, depending on calculation method), which cannot explain the dramatic enhancement of insulin secretion (Fig. 1*C*). Moreover, total pancreatic insulin content was identical in βLKB and control mice in both the fasting and fed states (Fig. 1*D*), further suggesting that increased insulin secretion is not due to modulation of insulin content.

To characterize insulin secretion in greater detail, we analyzed GSIS in islets isolated from βLKB and control mice and normalized the measurements to DNA content. Similarly to our *in vivo* measurements in perfused pancreata (23), perifused βLKB islets secreted normal levels of insulin in low glucose but showed greatly enhanced insulin secretion upon a shift to high glucose (Fig. 1*E*). The temporal pattern of secretion was normal, with more insulin released in both the first and second phases. When KCl was added to obtain maximal secretion, βLKB islets secreted 2-fold more insulin than controls. These results rule out mechanisms of secretion that may operate *in vivo* only, for example more rapid or efficient secretion to blood due to altered cell polarity. Rather, they demonstrate that persistent enhanced secretion in βLKB mice is β cell autonomous and point to a mechanism distal to plasma membrane depolarization.

##### K_ATP_ and Calcium Channels Are Required but Not Sufficient for Enhanced Insulin Secretion in βLKB Mice

**FIGURE 2.**
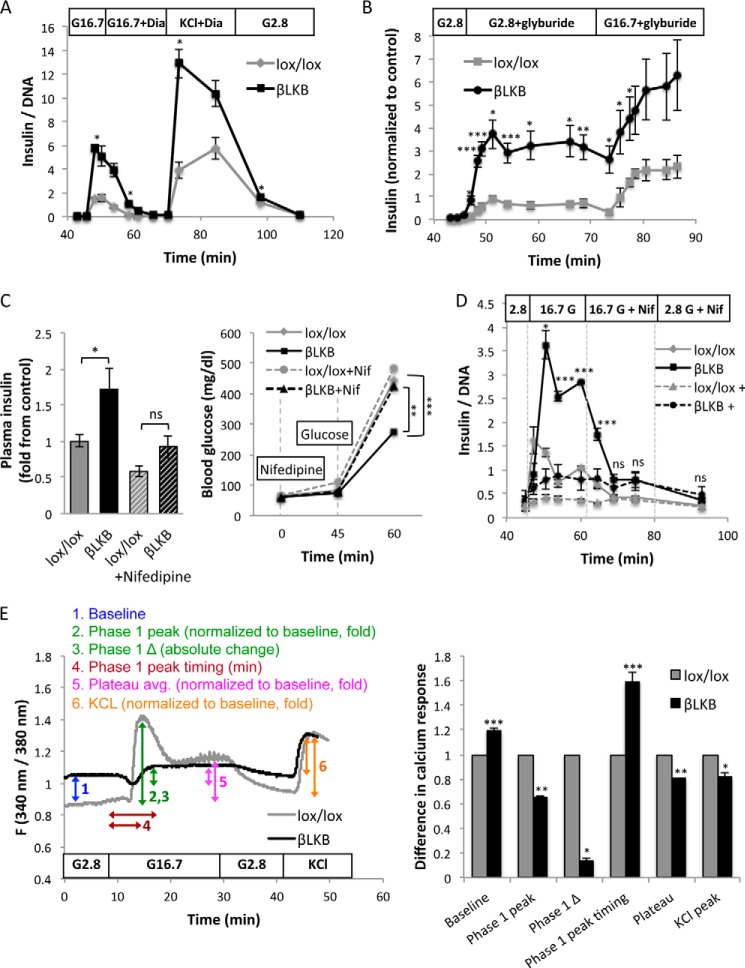
**Insulin secretion in LKB1-deficient β cells is *K*_ATP_-dependent.** *A*, insulin levels during an islet perifusion assay. Switching medium from 2.8 to 16.7 mm glucose (G16.7) causes higher secretion in LKB1-deficient islets. Diazoxide (*Dia*) (100 μm) abolishes secretion in both control and mutant islets, and further addition of 30 mm KCl triggers a second peak of secretion. Data represent the mean of 2 groups of islets taken from different mice at age of 2.5 months. *B*, insulin secretion during islet perifusion with glyburide. Glyburide (1 μm) triggers dramatically more insulin secretion from LKB1-deficient islets in either low or high glucose. Data represent mean of data from three (control) and five (LKB1-deficient) mice. *C*, serum insulin and glucose levels after administration of nifedipine (*Nif*) to lox/lox and LKB1-deficient mice after an overnight fast. Nifedipine (10 mg/kg in 5% DMSO) was injected at time 0. Glucose was injected at 45 min. Glucose was measured at 0, 45, and 60 min, and insulin was measured at 60 min. Mice were 3–12 months old. *n* = 6–9 mice per group. *D*, glucose-stimulated insulin secretion from perifused islets treated with nifedipine. *Dashed lines*, nifedipine was added before high glucose. *Solid lines*, nifedipine was added 15 min after the addition of high glucose. In both cases no significant difference was observed between lox/lox and βLKB islets in the presence of nifedipine. Statistical significance is shown for the experiment where nifedipine was added after high glucose. *E*, *left*, representative plots of calcium influx after glucose stimulation of wild type and βLKB islets. Islets were perifused with KRB buffer containing 2.8 or 16.7 mm glucose or 30 mm KCl as indicated. Intracellular calcium is calculated by the ratio of emission at 340- and 380-nm wavelengths using Fura-2 dye. Each plot represents the average ratio of 8–20 islets taken from one mouse. *Right*, calculation of 6 parameters of calcium response in lox/lox and βLKB islets, based on the calcium plots. Mice were 6 months old, *n* = 3 per genotype. *, *p* < 0.05; **, *p* < 0.01; ***, *p* < 0.005; *ns*, *p* > 0.05.

To clarify how βLKB islets secrete more insulin in response to glucose, we perturbed steps in the triggering pathway for insulin secretion. Treatment with diazoxide, a *K*_ATP_ channel opener, completely abolished glucose-stimulated secretion in perifused βLKB islets (Fig. 2*A*). This suggested that closure of *K*_ATP_ channels and membrane depolarization are essential for enhanced secretion in the mutants. However, when channels were forced to open with diazoxide and KCl was added, βLKB islets secreted more insulin than controls, consistent with the findings in Fig. 1*E*. Thus in the face of a similar degree of membrane depolarization, βLKB islets secrete more insulin, indicating enhancement of secretion at a distal step of the pathway (Fig. 2*A*). Furthermore, treatment with glyburide (forcing the closure of *K*_ATP_ channels) led to higher insulin secretion from cultured βLKB islets both at basal (2.8 mm) and stimulating (16.7 mm) glucose concentrations (Fig. 2*B*). In addition, *in vivo* administration of a high dose of glyburide boosted plasma insulin levels to a higher degree in βLKB mice compared with controls (data not shown). This further indicates a component boosting GSIS downstream to membrane depolarization.

We next treated mice with nifedipine, an inhibitor of voltage-gated calcium channels. Under these conditions, serum insulin levels after glucose injection were dramatically and equally reduced in βLKB and control mice (Fig. 2*C*), and blood glucose levels rose to equally high levels in mutants and controls. Similar results were obtained when we treated perifused islets with nifedipine and measured glucose-stimulated insulin secretion. As shown in Fig. 2*D*, nifedipine dramatically reduced insulin secretion in both LKB1-deficient and wild type islets. These results indicate that L-type calcium channels are required for insulin secretion in βLKB islets.

To better understand calcium dynamics in βLKB islets, we measured intracellular calcium flux in isolated islets exposed to different glucose levels. We incubated islets at different glucose concentrations and imaged them in the presence of Fura-2AM, a sensitive indicator for intracellular free calcium ions. Surprisingly, βLKB islets had abnormally high levels of Ca^2+^ at low glucose (presumably not reaching the threshold needed for a measurable increase in insulin secretion). More importantly, LKB1-deficient islets largely failed to enhance calcium in response to high glucose. Calcium levels in βLKB islets did rise upon treatment with KCl, suggesting that forced membrane depolarization does mobilize calcium in mutant cells (Fig. 2*E*). Further analysis using the fluo-2 dye confirmed that the amplitude of the response was a decrease in LKB1-deficient cells (not shown) and demonstrated that the fraction of responding β cells was decreased in βLKB islets (lox/lox, 66.9 ± 6.1% cells responding; βLKB, 28.7 ± 6.1% responding; *p* < 0.01). Thus, βLKB islets have a defect in glucose-stimulated calcium entry, likely at a step upstream to membrane depolarization. Nonetheless, and remarkably, this defect does not interfere with enhanced GSIS.

##### Altered Responses to Succinate and Leucine in βLKB Islets

**FIGURE 3.**
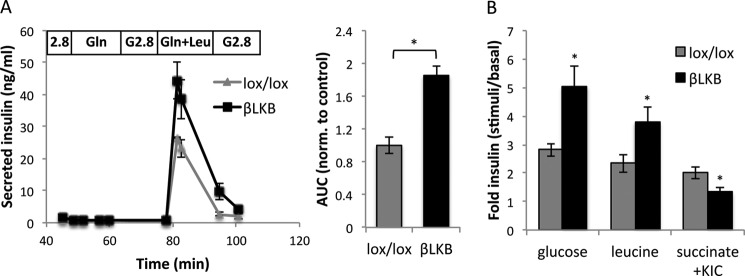
**Effects of glutamine, leucine, and succinate on insulin secretion.** *A*, islet perifusion assay with glutamine (*Gln*) or glutamine + leucine (*Gln*+*Leu*) at low glucose. Each plot represents the mean of two different mice. Mice are 6 months old. *Right*, mean of area under the curve (*AUC*) calculated for the perifusion plots. *B*, insulin secretion in response to glucose (16.7 mm), leucine (10 mm, in the presence of 3 mm glucose), and methyl succinate (10 mm) plus α-ketoisocaproate (*KIC*; 2 mm) in lox/lox and βLKB islets using static incubation. Mice were 4 months old. Data represent the mean of three or four independent experiments. *, *p* < 0.05.

Glucose triggers calcium entry and insulin secretion via its oxidative phosphorylation in the mitochondria and the generation of ATP, leading to closure of *K*_ATP_ channels. We hypothesized that the defect in glucose-induced calcium dynamics reflected a defect in mitochondrial metabolism. To test this idea, we examined insulin secretion from βLKB islets in response to mitochondrial fuels. Perifusion of islets with glutamine (which can enter the TCA cycle through activity of glutamate dehydrogenase) did not increase insulin secretion at low glucose levels in both βLKB and control islets. However, glutamine combined with leucine, an activator of glutamate dehydrogenase (40), did trigger insulin secretion at low glucose (Fig. 3*A*), and this response was stronger in βLKB islets compared with controls. Surprisingly, control experiments where leucine was added alone revealed a similar increase in insulin secretion. The response to leucine alone was higher in βLKB than in control islets, and the magnitude of the effect was similar to that seen in response to glucose (Fig. 3*B*).

We then treated islets with another classic mitochondrial substrate, monomethyl succinate (added to islets together with low levels of α-ketoisocaproate (41)). Strikingly, succinate did induce insulin secretion in control islets but failed to do so in βLKB islets.

These results support the idea that βLKB islets have a defect in mitochondrial-induced calcium entry and insulin secretion. In addition, they suggest that βLKB islets are more sensitive to a mitochondrial-independent secretory stimulation by leucine.

##### Lkb1 Is Essential for Normal Oxidative Mitochondrial Function

**FIGURE 4.**
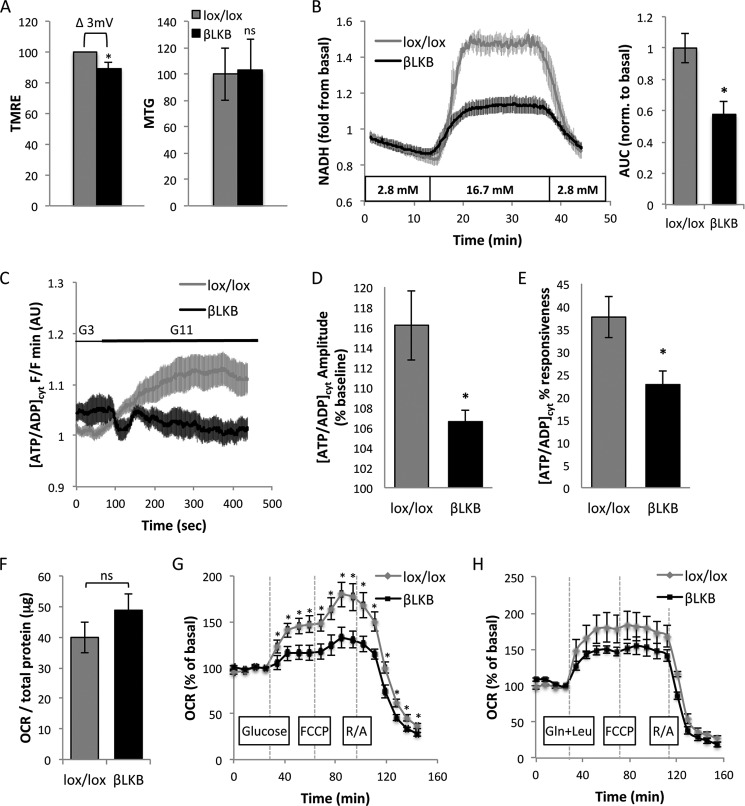
**Lkb1 deletion in β cells disrupts mitochondrial function.** *A*, quantification of fluorescence intensity of TMRE (*left*) and MitoTracker Green (*MTG*, *right*) in islet cells from lox/lox and βLKB mice. Mice were 3 months of age, 2 months post-tamoxifen injection. The graph presents the mean of five mice in each genotype. 20–30 fields were imaged and averaged per mouse. The difference in fluorescence intensity of TMRE is translated to Δ 3 mV between lox/lox and βLKB cells. *B*, NAD(P)H production in response to 16.7 mm glucose. The plots represent the mean of NAD(P)H-derived fluorescence intensity in whole islets from lox/lox (*n* = 4) and βLKB (*n* = 4) mice measured by UV autofluorescence. Islets were perifused with 2.8 mm glucose for 12 min then with 16.7 mm for 15 min and back to 2.8 mm. Mice were 6 months old, 5 months post-tamoxifen injection. *Right*, mean of area under the curve (*AUC*) of the NAD(P)H plots. *, *p* < 0.05. *C*, cytosolic ATP/ADP ratio changes in islets in response to glucose. Glucose was changed during the experiment from 3 mm (G3) to 11 mm (G11). Note the significantly impaired response in βLKB1 islets. *AU*, arbitrary units. Data are from three wild type and three βLKB mice. *D*, amplitude of responsiveness presented in *C. E*, ATP/ADP ratio presented as % of islets responsive to glucose. *, *p* < 0.05. *F*, basal OCR measured by Seahorse XF24 analyzer in the presence of 2.8 mm glucose. *ns*, *p* > 0.05. *G*, OCR measured over time. Data are presented as -fold induction from basal OCR presented in *F*. Each plot represents the mean of 7 wells with 50 islets each from wild type (*n* = 3) or βLKB (*n* = 4) mice. Mice were 2.5 months old. Compounds injected at indicated times were glucose (20 mm), FCCP (1 μm), and rotenone plus antimycin A (*R/A*, 5 μm each). *H*, OCR in the presence of glutamine and leucine (*Gln*+*Leu*). Protocol is as described in *G*. Each plot represents the mean of 6 or 7 wells with 50 islets from wild type (*n* = 5) or βLKB (*n* = 4) mice. Mice were 6 month old. The assay were performed on Pdx1-CreER^TM^;LKB1 ^lox/lox^ mice, except for ATP measurements (*C–E*) that were performed on Ins1-Cre;LKB1^lox/lox^ mice aged 10 weeks.

The failure of succinate to increase insulin secretion and the defect in glucose-induced calcium mobilization prompted us to examine mitochondrial function of βLKB islets. We first analyzed mitochondrial membrane potential in cultured dissociated islet cells from βLKB and control mice using the mitochondrial membrane potential-sensitive dye TMRE. Measuring fluorescence intensity of TMRE by confocal imaging showed reduced TMRE in βLKB islet cells (2 months post deletion of LKB1). Importantly, co-staining with the mitochondrial membrane potential-non-sensitive mitochondrial mass dye, MitoTracker Green, showed no difference in βLKB islet cells (Fig. 4*A*). Using a modified version of the Nernst equation (34), we translated the reduction in TMRE fluorescence intensity to Δ 3 mV in βLKB islets cells. Thus, deletion of LKB1 causes relative depolarization of β cell mitochondria.

Measurements of NAD(P)H also indicated a mitochondrial defect in βLKB islets. NAD(P)H levels were dramatically increased in control islets perifused with high glucose and returned to baseline upon transfer to low glucose. By contrast, the NAD(P)H response to glucose was significantly blunted in βLKB islets (5 months post LKB1 deletion), indicating a defect in mitochondrial glucose metabolism (Fig. 4*B*). Furthermore, by using the ATP/ADP ratio probe, Perceval (36), we showed that the glucose-induced rise in ATP/ADP ratio (which is the direct mediator of *K*_ATP_ channel closure, calcium entry, and insulin secretion) was prominent in controls but reduced in mutant islets (Fig. 4*C*). The effect was evident both by a reduced amplitude of response (Fig. 4*D*) and by a smaller fraction of cells that responded to stimulus (Fig. 4*E*).

We also assessed the rate of oxygen consumption in βLKB islets using the Seahorse XF analyzer. OCR was measured in low glucose, then in response to high glucose, and then after adding the mitochondrial uncoupler FCCP to force maximal oxygen consumption. Although basal OCR was similar in control and βLKB islets (Fig. 4*F*), oxygen consumption at high glucose was lower in βLKB islets (Fig. 4*G*). Even forced uncoupling using FCCP elicited reduced OCR in βLKB islets compared with controls. Blocking the respiratory chain with rotenone and antimycin resulted in complete abolishment of oxygen consumption in both control and βLKB islets, excluding non-mitochondrial oxygen consumption in either genotype. Lastly, we tested the effect of leucine and glutamine on OCR. The response of mutant islets to these amino acids was similar or reduced compared with wild type islets (Fig. 4*H*), supporting the idea that they boost insulin secretion in Lkb1-deficient islets via a mitochondria-independent mechanism. Together, these studies reveal a dramatic functional defect in the mitochondria of Lkb1-deficient β cells.

##### Deletion of Lkb1 Leads to Mitochondrial Destruction

**FIGURE 5.**
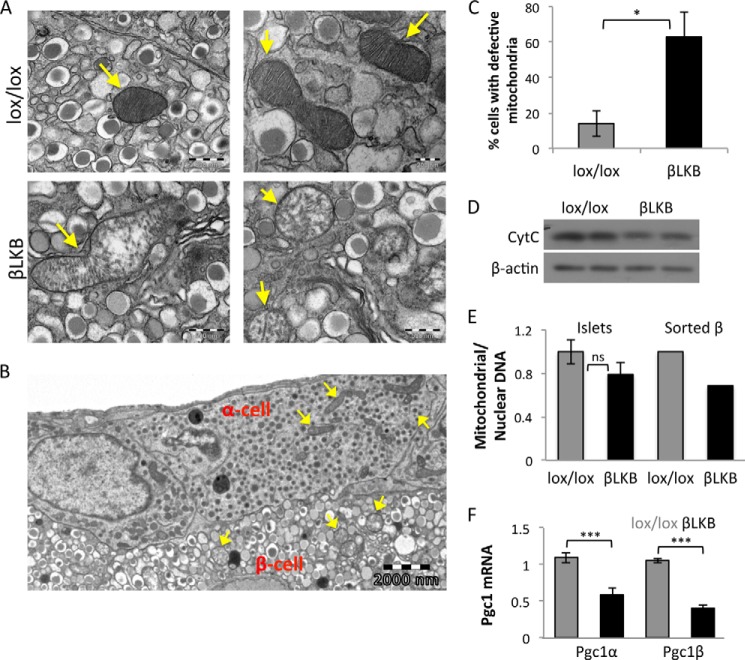
**LKB1 is essential for mitochondrial integrity.** *A*, representative transmission electron micrographs of pancreatic sections showing mitochondria (*arrows*) in β cells in lox/lox and βLKB mice. Mice were 10 months old, 9 months after tamoxifen injection. *B*, a section from an islet of an LKB1-deficient mouse showing intact mitochondria in two α-cells. *Arrows* point to mitochondria. *C*, quantification of the percentage of β cells with defective mitochondria (swollen mitochondria or lack of cristae) as determined by analysis of EM images. *, *p* < 0.05. *D*, Western blot of cytochrome *c* protein normalized to actin on islets from two wild type mice and two βLKB mice. Mice were 5 months old. *E*, mitochondrial DNA copy number measured by the ratio between mitochondrial DNA and nuclear DNA from lox/lox and βLKB whole islets (*left*) or sorted β cells (*right*) using qPCR. For whole islets, data represent the mean of DNA ratio (cytochrome *b*/Aprt) from 4–5 mice at 4 and 10 months of age. For sorted β cells, data represent the ratio of DNA (cytochrome *b*/L1) pooled from 4 mice per genotype at 1 year of age. *F*, PGC1α and PGC1β mRNA levels quantified by RT-PCR from wild type and βLKB islets normalized to actin. Data represent the mean ± S.E. of 4 mice at age of 4.5 months. ***, *p* < 0.005.

To understand the basis for the functional defect in mitochondria of βLKB β cells, we examined mitochondrial structure at high resolution using transmission electron microscopy. Islets of control islets presented with a typical pattern of mitochondria, including the fine structure of cristae (Fig. 5*A*). Islets of βLKB mice, examined 3–10 months after deletion of LKB1, revealed a strikingly different pattern of mitochondria. Mitochondria in mutant β cells were swollen and absent of cristae. The effect was specific to β cells, as adjacent α cells in the same sections had the normal appearance of mitochondria (Fig. 5*B*). The latter observation also rules out artifacts related to fixation and processing of tissue. The mitochondrial defect was found in 60% of β cells in βLKB islets (*n* = 6 mice, 15–20 cells examined per mouse) compared with ∼12% of mitochondria that had such appearance in wild type islets (Fig. 5*C*). In affected β cells, all mitochondria were apparently disrupted. This suggests a cell autonomous, all or none effect, occurring in the LKB1-deleted β cells and sparing cells that have escaped cre-mediated deletion. We have not observed other ultra-structural alterations in βLKB islets beyond the previously reported distended endoplasmic reticulum (42). In particular, we tested whether LKB1-deficient islets had more insulin granules docked at the plasma membrane (up to 200 nm from the membrane) as a potential indication for enhancement in distal release but found no difference between wild type and mutant islets (data not shown).

Assessment of the mitochondrial protein cytochrome *c* by Western blotting revealed a moderate decrease in βLKB islets (Fig. 5*D*). Quantitative PCR revealed a trend for a reduction in mitochondrial DNA (ratio of cytochrome *b* DNA to Aprt DNA) that did not reach statistical significance, possibly due to the presence of non-β cells as well as non-recombined β cells in the islet preparations. Indeed, when mitochondrial DNA was measured in sorted β cells, we observed an ∼30% reduction in mitochondrial/nuclear DNA ratio in β cells isolated from βLKB mice (Fig. 5*E*). Consistent with the moderate decrease in mitochondrial protein and DNA seen in βLKB islets, there was no difference in the intensity of fluorescence when control and βLKB islets were treated with the mitochondrial mass marker MitoTracker Green (Fig. 4*A*).

Finally, we sought to identify the molecular basis for degeneration of mitochondria in LKB1-deficient β cells. PGC1α and PGC1β are central nuclear transcriptional regulators of mitochondrial biogenesis (43, 44) that were shown in other systems to be regulated at the mRNA level by phosphorylated AMPK, a central target of LKB1 (45, 46). Quantitative PCR analysis revealed a 2-fold reduction of PGC1α and PGC1β mRNA in βLKB islets compared with controls (Fig. 5*F*), potentially explaining the mitochondrial defect via a LKB1-AMPK-PGC1 axis.

##### Evidence for Cell Autonomous Effects of LKB1 on Insulin Secretion and Energy Metabolism

**FIGURE 6.**
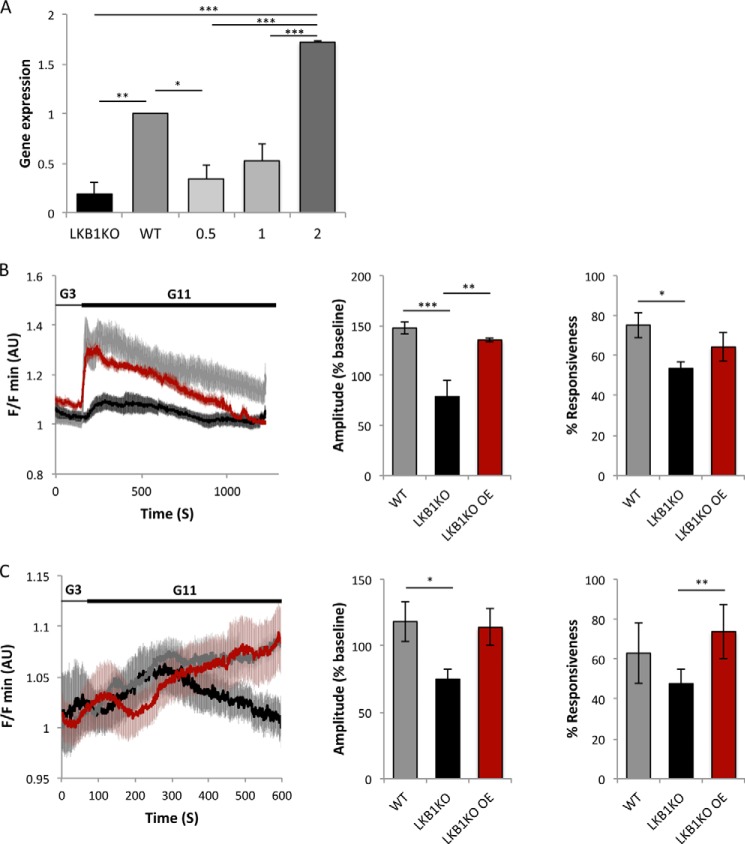
***In vitro* rescue of LKB1-deficient β cells.** *A*, LKB1 mRNA levels measured by qRT-PCR in cultured wild type and βLKB islets, 48 h after infection with adenoviruses encoding wild type LKB1. *n* = 3 mice/condition. Cells were infected at the multiplicity of infection indicated. *, *p* < 0.05; **, *p* < 0.01; ***, *p* < 0.005. *B*, rescue of glucose-stimulated calcium influx in LKB1-deficient islets. Both the amplitude (*center*) and the fraction of responding cells (*right*) returned to normal upon LKB1 re-expression. *n* = 3 mice per genotype, 9–12 islets per genotype. *AU*, arbitrary units. *C*, rescue of glucose-stimulated [ATP/ADP]_cyt_ rise in LKB1-deficient islets. Both the amplitude (*center*) and the fraction of responding islets (*right*) are improved upon LKB1 re-expression. *n* = 5 mice per genotype, 9–15 islets per genotype. *, *p* < 0.05; **, *p* < 0.01.

The deletion of LKB1 in β cells *in vivo* is incomplete, as in most tamoxifen-inducible mouse systems. Therefore, the phenotypes analyzed here could in principle reflect either a cell autonomous, direct effect of LKB1 deletion in β cells or, alternatively, a compensatory effect in wild type β cells. We showed before that LKB1 deletion leads to improved glucose tolerance *in vivo* as early as 1 week after tamoxifen injection (23), strongly suggesting that the underlying mechanism is a direct enhancement of insulin secretion from LKB1-deficient β cells. In addition, a recent paper has documented enhanced secretion in min6 cells with LKB1 knockdown (47), further supporting this conclusion. To examine whether the energy metabolism phenotype (defective mitochondrial function) also acts in a cell autonomous manner, we reintroduced wild type LKB1 via adenoviral infection to cultured LKB1-deficient islets. Within 48 h of infection, LKB1 levels in mutant islets were restored (Fig. 6*A*), and both calcium influx and ATP levels were restored to near-normal levels (Fig. 6, *B* and *C*). These results indicate that the defects in energy metabolism in LKB1-deficient islets are a direct effect of LKB1 loss in β cells rather than an indirect compensatory mechanism.

## Discussion

We show here two opposing effects of LKB1 deficiency in pancreatic β cells. The absence of LKB1 causes dramatic functional and structural defects in the mitochondria of β cells. On the other hand, glucose-stimulated insulin secretion is enhanced in LKB1-deficient β cells, *in vivo* and in isolated islets, and persists for at least 16 months after deletion of the gene. Moreover, mice with LKB1-deficient β cells were shown to resist high fat diet-induced glucose intolerance (25). Our findings suggest that LKB1 deficiency stimulates a robust amplifying pathway, which overrides the defects in the classical triggering pathway of insulin secretion relying on oxidative metabolism of glucose to ATP.

### 

#### 

##### Regulation of Glucose-stimulated Insulin Secretion by LKB1

LKB1 deficiency causes a dramatic enhancement of GSIS (this work and Refs. 23, 24, and 25), but this net effect reflects the integration of multiple phenotypes acting in opposing directions. Previous studies showed that two LKB1 phosphorylation targets, SIK2 and SAD-A, positively regulate insulin secretion, such that in their absence there are secretion defects (26, 27). As for AMPK, its effect on GSIS has remained controversial (48–50), perhaps because its own downstream effectors act in opposing directions on the secretory machinery, potentially in a context-dependent manner. In the present work we report that LKB1 is essential for mitochondrial function in β cells (see below). This adds a major hurdle to GSIS in LKB1-deficient β cells. Indeed, mitochondrial oxidation of glucose and the TCA cycle fuel succinate is defective, rendering the classic triggering pathway inactive in LKB1-deficient β cells. In light of these defects, the dramatic enhancement of GSIS in mutants must involve powerful pro-secretion mechanisms that override the multiple intrinsic defects.

Our work provides molecular evidence on how a lack of LKB1 in β cells improves GSIS. First, the improvement cannot be merely a consequence of increased β cell mass or pancreatic insulin content. We found that, in tamoxifen-deleted strains, insulin content in βLKB pancreata was not significantly increased, and β cell mass was only moderately increased to a degree that cannot explain the robust increase in secretion (we note, however, that increases in β cell mass may play a more significant role in enhanced *in vivo* GSIS in the in utero-deleted models (24, 30). The observation of increased GSIS in perifused islets (with normalization to DNA content in the sample) further suggests that insulin secretion is enhanced in a cell-autonomous manner. Second, reversed β cell polarity is unlikely to be the cause for enhanced GSIS. Although altered polarity of βLKB cells could theoretically promote insulin secretion in response to glucose (23), the fact that LKB1-deficient islets secrete more insulin *in vitro*, where blood vessels and the circulation play no role, argues against this possibility.

Even though the classic triggering pathway (51) is defective in LKB1-deficient β cells, some level of calcium entry via voltage-gated calcium channels is essential for insulin secretion in these cells, as prevention of membrane depolarization or inhibition of calcium channels eliminated secretion in the mutants. These findings point to a distal component as the key determinant of enhanced GSIS in LKB1 mutants. Although the exact molecular mechanism remains elusive, an important hint might be the responsiveness of LKB1-deficient β cells to leucine. Leucine is normally considered a secretagogue via its activation of glutamate dehydrogenase in the mitochondria, supplying α-ketoglutarate to the TCA cycle (40, 52). However, this is unlikely the mechanism of action in LKB1-deficient β cells that contain dysfunctional mitochondria and do not respond to succinate. Alternatively, leucine can also be metabolized by transamination. This may increase the concentration of glutamate in β cells, which has been shown recently to boost insulin secretion via intracellular activity on the exocytosis machinery (53–55), although this has been controversial (56). Interestingly, our transcriptome analysis of LKB1-deficient β cells has revealed major alternations in pathways related to glutamine and glutamate synthesis and processing (30). Specifically, cytosolic aminotransferases (Bcat1 and Tat) were significantly up-regulated (by 4.5- and 1.7-fold, respectively) in LKB1-deficient islets. We thus speculate that increased levels of intracellular glutamate in LKB1-deficient β cells primes for more effective glucose-stimulated insulin secretion, requiring less calcium to trigger release. Consistent with this idea, we measured increased glutamate levels in LKB1-deficient islet cells (lox/lox, 0.02 ± 0.002 nmol glutamate/μg of protein; βLKB, 0.03 ± 0.002 nmol of glutamate/μg of protein; *p* < 0.05). Importantly, our previous study (30) also demonstrated a substantial up-regulation of genes involved in glutamate signaling in βLKB1 islets and increased responsiveness to exogenously applied glutamate receptor agonists. It is, therefore, tempting to speculate that an action of intracellular glutamate on secretory granules themselves (57) or enhanced release of glutamate into the extracellular space and agonism at cell surface glutamate receptors may contribute to enhanced insulin secretion in LKB1 null β cells.

A very recent paper from Screaton and co-workers (47) also examined β cell function in the absence of LKB1. Their findings are in general agreement with ours, in that GSIS is enhanced in mutant β cells despite a mitochondrial defect. However, their proposed mechanism involves glutamine to citrate metabolism as well as increased activity of acetyl-CoA carboxylase 1 (ACC1). In addition, Fu *et al.* (47) have described increased insulin granule docking in LKB1 mutant β cells, which was not observed in the current study. More work is needed to test these ideas and to identify the precise molecular mechanisms that enhance GSIS in LKB1-deficient β cells, including the relevant LKB1 phosphorylation target(s). Regardless, our findings highlight the relative importance of distal components in the insulin secretion pathway (amplifying signals) compared with the classic triggering pathway.

These findings may have implications beyond the understanding of LKB1 biology. Enhanced glucose-stimulated insulin secretion is a common compensatory mechanism in β cells under an increased workload (*e.g.* insulin resistance), but compensation often turns into decompensation, resulting in the development of type 2 diabetes (58). This biphasic behavior of β cells is not fully understood but appears to be a universal phenomenon seen not only in type 2 diabetes but also in congenital hyperinsulinism (59–61) and in different rodent models such as leptin receptor deficient mice (db/db), (62), β cell-specific deletion of TSC2 in mice (39), and *Psammomys obesus* exposed to a high calorie diet (63). LKB1-deficient β cells are remarkable not only due to the enhancement of GSIS in the face of degenerated mitochondria (and virtually absent triggering pathway for secretion) but are also due to the persistence of the phenotype. Even 16 months after LKB1 deletion, βLKB mice showed greater insulin secretion and consequently improved glucose tolerance, with no evidence for decompensation. This shows that β cells, even in the face of mitochondrial dysfunction and additional defects in the secretion machinery, can still be manipulated to boost insulin secretion over long periods of time. A drug that mimics the pro-secretion activity of LKB1 deficiency can theoretically cause a long term improvement of β cell function in type 2 diabetes patients.

##### Regulation of Mitochondrial Structure and Function by LKB1

We found that LKB1 deficiency in β cells leads to mitochondrial degeneration, as illustrated by electron microscopy. This is accompanied by functional defects in oxidative metabolism as well as in downstream signaling such as calcium entry. Thus LKB1 is essential for the maintenance of mitochondria in adult β cells. We propose that the underlying mechanism is control of mitochondrial biogenesis via PGC1, a known target of phospho-AMPK (45, 46) and a key regulator of mitochondria (43, 44). In support of this hypothesis, the levels of PGC1α and PGC1β were significantly down-regulated in LKB1-deficient β cells. We note, however, that ectopic expression of PGC1 was shown before to cause islet failure and impaired insulin secretion (64). The different phenotype in our study likely results from the pleiotropic effect of LKB1in β cells. Alternatively, LKB1-deficient cells may have defects in mTOR-controlled mitophagy (65, 66), leading to the accumulation of defective mitochondria. Previous studies have shown that LKB1 is an important regulator of mitochondrial metabolism (although ultrastructural defects as in our study were not reported), but the effect was proposed to be unique to hematopoietic stem cells (20–22). Our work suggests that LKB1 is universally important for mitochondrial homeostasis. Considering the established role of LKB1 as a tumor suppressor, this notion provides a new mechanism for aerobic glycolysis in cancer (the Warburg effect). Interestingly, although current thinking describes aerobic glycolysis as a normal feature of rapidly proliferating cells (67), Otto Warburg himself was convinced that cancer cells use aerobic glycolysis due to defects in their mitochondria (68). To the best of our knowledge LKB1 is the only tumor suppressor whose absence indeed damages the mitochondria. We found no evidence for enhancement of glycolysis in LKB1-deficient β cells, although in other systems it was demonstrated that LKB1 loss does increase glucose uptake and glycolysis via the activity of HIF1, a master regulator of glycolytic gene expression (69, 70).

In summary, we show here that LKB1 is essential for the maintenance of mitochondria in adult pancreatic β cells, a function that is likely exerted by LKB1 in multiple cell types. Although the mitochondrial defect in LKB1-deficient β cells acts to impair fuel-stimulated insulin secretion, surprisingly, the net effect of LKB1 deficiency in β cells is a persistent improvement of glucose-stimulated insulin secretion and consequently glucose tolerance, likely via effects on distal steps in the secretion machinery. Unraveling the molecular mechanism underlying increased insulin secretion in LKB1-deficient β cells may open new therapeutic approaches for type 2 diabetes.

## Author Contributions

A. S. and Y. D. conceived and coordinated the study. Y. D., A. S., L. P., G. A. R., G. L., and B. G. wrote the paper. A. S., Z. G., N. T., S. S., D. J. H., and J. D. W. performed and analyzed the experiments. N. B. contributed reagents. N. T. and S. S. contributed to the preparation of the figures. All authors reviewed the results and approved the final version of the manuscript.

## Footnotes

6 The abbreviations used are:

LKB1: liver kinase B1
AMPK: adenosine monophosphate activated protein kinase
GSIS: glucose-stimulated insulin secretion
KRBB: Krebs-Ringer buffer
TMRE: tetramethylrhodamine, ethyl ester
FCCP: carbonyl cyanide 4-(trifluoromethoxy)phenylhydrazone
OCR: oxygen consumption rate.
